# New Zealand consumers’ perceptions of private insurance for pharmaceuticals

**DOI:** 10.1186/2193-1801-3-587

**Published:** 2014-10-08

**Authors:** Rajan Ragupathy, Zaheer-ud-Din Babar, Wasif Mirza, Mitali Daiya, Himesh Chandra, Ali Yousif, Maninder Girn

**Affiliations:** Pharmacy Services, Waikato Hospital Pembroke Street, Hamilton, 3210 New Zealand; School of Pharmacy, Faculty of Medical and Health Sciences, University of Auckland, Park Road, Auckland, 1023 New Zealand

## Abstract

Private insurance plays a minor role in paying for pharmaceuticals in New Zealand, despite controversy about access through the public health system. The present study examines New Zealand consumers’ perceptions of private insurance for pharmaceuticals. A self-administered questionnaire was completed by 433 consumers at thirty pharmacies. The questionnaire included 18 questions on demographics, insurance status, perceptions of private insurance for pharmaceuticals and confidence in the public health system. Forty six percent of respondents had private health insurance. Respondents were more likely to have private health insurance as household income increased, and confidence in the public health system decreased. (Over two thirds of respondents were either confident or very confident in the public health system). Nineteen percent had private health insurance *for pharmaceuticals,* and the likelihood was not affected by household income or confidence in the public health system. Sixty one percent believed private insurance for pharmaceuticals would increase availability and affordability of pharmaceuticals. However, just over half were willing to pay for private insurance for pharmaceuticals. Of these, over two thirds were only willing to pay $20 per year or less. New Zealand pharmacy consumers’ willingness to pay for private insurance for pharmaceuticals is very low.

## Introduction

Private insurance pays for a mere 2% of pharmaceutical costs in New Zealand (The Organisation for Economic Co-operation and Development 2010a). In contrast, private insurance pays for 17% of outpatient pharmaceutical costs in France, and 30% in Canada and the United States (The Organisation for Economic Co-operation and Development 2010b). New Zealand’s per capita pharmaceutical expenditure is also lower than such countries. In 2010 this was 288 United States Dollars Purchasing Power Parity (US$ PPP), compared with 637 US$ PPP in France, 739 US$ PPP in Canada and 973 US$ PPP in the United States (The Organisation for Economic Co-operation and Development 2013).

New Zealanders pay for approximately 30% of pharmaceutical costs out-of-pocket, a figure which includes prescription charges, prescription medicines that are not publically funded, and non-prescription medicines. The proportion of out-of-pocket spending in New Zealand is higher than in France, Canada or the United States (The Organisation for Economic Co-operation and Development 2011). Increasing levels of private insurance for pharmaceuticals may therefore both supplement public pharmaceutical spending and reduce out-of-pocket expenditures. In the present study, we examine New Zealanders’ perceptions of private insurance coverage for pharmaceuticals.

One possible reason private insurance currently plays such a small role is that New Zealanders have access to a wide range of publically funded pharmaceuticals. All pharmaceuticals used in hospitals are provided free of charge to the patient. New Zealand also has a national list of several hundred publically funded cancer and outpatient pharmaceuticals (estimated at 471 chemical entities in 2007), which are listed in the Pharmaceutical Schedule. Over 85% of pharmaceuticals in the Schedule are fully subsidised, with most patients only paying a NZ $3 (US $2.2) prescription charge per three month supply of each pharmaceutical at the time of data collection. (The prescription charge increased to NZ $5 in 2013) (New Zealand Ministry of Health 2013; Aaltonen et al. 2010; Cumming et al. 2010). The remaining pharmaceuticals on the Schedule are partly subsidised, with patients paying the difference in addition to the prescription charge (Aaltonen et al. 2010; Cumming et al. 2010).

New Zealand’s strategy of providing a wide range of publically funded pharmaceuticals at low cost to patients depends heavily on the effectiveness of the New Zealand Pharmaceutical Management Agency (PHARMAC). PHARMAC has been held up as an example of how to increase access to pharmaceuticals while containing pharmaceutical costs (Cumming et al. 2010; Braae et al. 1999). PHARMAC manages a capped yearly budget for all pharmaceuticals on the Schedule. This includes conducting clinical and cost effectiveness evaluations of new pharmaceuticals that manufacturers want listed on the schedule, carrying out price negotiations with manufacturers, and setting the conditions under which pharmaceuticals listed on the Schedule will be subsidised (The New Zealand Ministry of Health 2014; The New Zealand Pharmaceutical Management Agency 2013). PHARMAC also negotiates national prices for many hospital pharmaceuticals, though the assessment and prioritisation of hospital pharmaceuticals was done individually by the country’s 20 District Health Boards (DHBs) at the time of data collection. The New Zealand Government has recently expanded PHARMAC’s role to include the assessment and prioritisation of hospital pharmaceuticals, and eventually also medical devices (Tordoff et al. 2005; Tordoff et al. 2008; Ryall 2013). These changes are likely to make PHARMAC an increasingly dominant part of New Zealand’s public health system.

However, PHARMAC is also arguably the most controversial part of New Zealand’s public health system. It has faced criticism for denying New Zealanders timely access to expensive but potentially life-saving pharmaceuticals (Isaacs et al. 2007). A report to the Government on access to high cost and highly specialised pharmaceuticals concluded that New Zealanders’ access to these pharmaceuticals lagged behind that in many other Organization for Economic Co-operation and Development (OECD) countries. (McCormack et al. 2013). International comparisons have shown also shown that New Zealand’s access to newly launched pharmaceuticals lags behind countries such as Finland, Australia, the United Kingdom and the United States (Aaltonen et al. 2010; Ragupathy et al. 2012; Raftery 2008).

In light of these controversies and concerns about access, it is worth examining if New Zealand consumers are willing to pay for alternatives to the public health system. The high cost of many new pharmaceuticals (which can be tens or hundreds of thousands of dollars per year) mean that paying for non-publically funded medicines out-of-pocket is unrealistic for all except the most wealthy (Canadian Cancer Society 2009). Private insurance for pharmaceuticals as in countries such as France, Canada and the United States is a possible alternative.

## Methods

Ethics approval was obtained from the University of Auckland Human Participants Ethics Committee. The study is a survey of pharmacy consumers in Auckland, New Zealand. Copies of the survey instrument, participant information sheets, participant consent forms, pharmacist information sheets and pharmacist consent forms are available from the authors on request.

The survey was conducted using a self-administered questionnaire. The survey instrument was developed for this study. The initial draft of the questionnaire was pilot tested on 10 individuals (of different ages and ethnic backgrounds, and who had no specialist knowledge of the health system) to test for comprehensibility and validity. The questionnaire was modified based on this testing, and pilot tested on a further ten individuals. Following this testing, the questionnaire was modified slightly before being approved by all the authors.

The final questionnaire contained 18 questions, and was divided into four parts. These were: demographics (age, gender, ethnicity, occupation, education and household income), current health status (including use of medicines and spending on medicines), current insurance status, and perceptions of the public health system.

A list of all pharmacies in the Auckland region was obtained from the Pharmaceutical Society of New Zealand. A random number generator was used to draw 80 pharmacies from this list. (The investigational plan called for a survey of 30 pharmacies, but 80 were selected initially to account for those who might decline to take part). Initial contact was by telephone, and those pharmacies which were willing to take part were sent pharmacist information sheets and consent forms (with the option of again declining to take part once they had a chance to read these). Recruitment stopped when 30 pharmacies had given written consent to take part in the survey.

A study member was at each of the participating pharmacies from 10 AM to 5 PM Monday through to Friday. Every second customer was approached to take part, and if the customer declined to take part, the next available customer was approached. If the customer gave verbal consent to take part, they were then given the participant information sheet and consent form to read and sign before being given the questionnaire. The study member was on hand to explain any questions participants had difficulty with, but participants were also given the option of skipping any questions they didn’t want to answer.

Approximately 60% of those approached took part. There were 433 respondents in total (The characteristics of those who took part are in Table 1). SPSS statistical software was used to process the data.

**Table 1:** **Characteristics of survey participants**

		Frequency	Percent
**Gender**	Male	172	39.7
	Female	261	60.3
**Age**	18-25	58	13.3
	26-35	92	21.1
	36-45	91	20.8
	46-55	79	18.1
	56-65	53	12.1
	65+	64	14.6
**Ethnicity**	Maori or Pacific Islander	65	14.8
	Asian	40	9.1
	Indian	40	9.1
	European	295	67.0
**Occupation**	Self Employed	10	2.4
	Unemployed	13	3.1
	Professional	162	38.1
	Technician / Trade worker	40	9.4
	Retail and administration	56	13.2
	Labourer	20	4.7
	Community and personal service worker	16	3.8
	Student	45	10.6
	Retired	47	11.1
	Housewife	16	3.8
**Education**	None	5	1.1
	Primary School	8	1.8
	Secondary School	149	33.9
	Tertiary Education	184	41.8
	Post Graduate	94	21.4
**Household income**	Less than 10,000	32	7.8
	10,000 - 30,000	57	13.9
	30,001 - 50,000	79	19.3
	50,001 - 70,000	71	17.3
	70,001 - 90,000	66	16.1
	90,000 +	105	25.6
**Health in general**	Poor	13	2.9
	Fair	58	13.1
	Good	242	54.8
	Excellent	129	29.2
**Visits to Pharmacy**	Once a week or more	25	5.7
	Once a month	140	31.7
	Once every 3 months	172	38.9
	Once every 6 months or more	105	23.8

Female customers are over-represented among survey participants, compared to the general population of Auckland in the 2006 Census (Statistics New Zealand 2014a). The over-representation of female customers among survey respondents replicates that in a separate study of Auckland pharmacy customers (which used different methods of selecting participating pharmacies and respondents) (Babar et al. 2010), and may therefore reflect the underlying demographics of weekday daytime Auckland pharmacy customers.

The proportion of Asian (including Indian) respondents was similar to that of the Auckland population in the 2006 Census (Statistics New Zealand 2014a). Maori and Pacific respondents are under-represented compared to the Auckland population, and this may reflect previous findings that Maori and Pacific people are more likely to forgo collecting prescriptions (and by implication visiting a pharmacy) because of cost (Jatrana et al. 2011). Conversely, Europeans were over represented. It should be noted matching ethnicity precisely to the Census is difficult, as the Census allows the selection of multiple ethnic identities, and includes ‘New Zealander’ as an option (Statistics New Zealand 2014b).

The age distribution of survey respondents was similar to that of the general Auckland population. Similarly, workforce participation characteristics (employed, unemployed, and those not in the labour force such as students, retirees and housewives) was similar to the general Auckland population (Statistics New Zealand 2014a).

## Results

### Private insurance coverage

Forty six percent of respondents had private health insurance, of whom 42% (19% of all respondents) had private health insurance coverage for pharmaceuticals. Eighty four percent of those with private insurance coverage paid for the insurance themselves or had the insurance paid for by a family member, while 16% had the insurance paid for by an employer.

Sixty five percent believed private health insurance should be subsidised by the government, and 72% stated that they would be more likely to purchase private health insurance if it were subsidised.

Chi square analysis showed none of age, gender, ethnicity or level of education had a statistically significant effect on whether respondents had private health insurance. Similarly, the number of visits to a pharmacy, average monthly spends at a pharmacy, or whether patients were on medicines that weren’t publically funded had no effect on whether respondents had private insurance.

The only factors that had significant effects on whether respondents had private health insurance or not were household income and confidence in the public health system. As household income increased, respondents were more likely to have private insurance coverage (chi square test p <0.001), odds ratio of 1.510 (95% CI 1.296 – 1.758). As confidence in the public health system increased, respondents were less likely to have private health insurance (chi square test p 0.044), odds ratio 0.633 (95% CI = 0.633 – 0.944).

### Beliefs regarding private insurance for pharmaceuticals

Sixty one percent of respondents believed having private insurance for pharmaceuticals would increase the availability and affordability of pharmaceuticals. However, only 52% were willing to pay extra for private insurance coverage of pharmaceuticals. Of these, 67% were only willing to pay $20 or less per year, and only 7% were willing to pay over $40 per year.

The most common reasons respondents cited for being willing to pay more for private coverage were convenience (any medicine that was needed would be covered), and as a precaution in case they developed serious illnesses later in life. The types of medicines patients were most likely to want covered by private insurance were those for cardiovascular disease (69% of respondents), cancer (67%), respiratory conditions (65%) and skin conditions (53%).

The most common reasons respondents cited for being unwilling to pay more for private insurance coverage were that the costs of insurance were too high, that they wouldn’t need it as they didn’t use medicines regularly, or that it would be more cost effective to pay the $3 prescription charges rather than the insurance premiums.

Chi square analysis show no statistically significant relationships between any of age, gender, ethnicity, level of education, household income or confidence in the public health system, and whether respondents were willing to pay for private insurance coverage for pharmaceuticals. Similarly, there was no relationship between the number of visits to a pharmacy, average spending in a pharmacy, or whether patients were on medicines that weren’t subsidised by the public health system, and whether respondents were willing to pay for private insurance coverage of pharmaceuticals.

### Confidence in the public health system

Eighty percent of respondents had experienced no problems with the public funding of their prescription pharmaceuticals in the previous 12 months. Eight percent had problems with funding changes (such as a change in which brand of a pharmaceutical was funded), 6% had experienced problems with the availability of pharmaceuticals, and 5% had problems with the affordability of pharmaceuticals.

Ten percent of respondents had delayed getting a prescription filled in the previous 12 months because of cost. Of these, 88% believed they would be more likely to have it filled if they had had a private insurance plan that covered pharmaceuticals (p <0.001).

Twenty seven percent of respondents were very confident in the public system, and 40% were confident. 15% were not confident in the public system, with the remainder being neutral.

## Discussion

The current study suggests that Auckland pharmacy consumers’ willingness to pay for private insurance coverage for pharmaceuticals is very low. Just over half of all respondents were willing to pay for such coverage, and of these 67% were only willing to pay $20 per year or less.

These results of course need to be interpreted in light of some limitations. This was a survey of ambulatory pharmacy customers, and as such the responses may not represent those of people who are severely ill or disabled, and therefore may not be able to visit a pharmacy. The survey was also done entirely in the city of Auckland, and as such the views of the respondents may not be totally representative of those in New Zealand as a whole. However, Auckland is home to a quarter of the New Zealand population, is ethnically and socioeconomically diverse, and draws internal migrants from all over New Zealand. New Zealand has a unitary system of Government (including national health, taxation and legal systems), which makes regional differences in the costs and benefits of having private insurance unlikely.

This reluctance to pay for private insurance may simply be a rational response to low prescription costs in New Zealand. The average prescription cost per patient per year was $19 in 2007, and had in fact declined from $24.30 (Cumming et al. 2010). As many respondents indicated, it may be more cost effective to pay prescription charges than insurance premiums.

Similarly, reluctance to pay for coverage may be due to consumers perceiving that their risk of being exposed to catastrophic pharmaceutical costs is relatively low. The public health system in New Zealand caps prescription charges at 20 charges per family per year, which means even a family with several members who need multiple medications a year would only pay a maximum of $60 per year if all pharmaceuticals are fully funded.(Cumming et al. 2010; The New Zealand Ministry of Health 2013a). Additional income and usage related safety nets can reduce prescription charges from $3 to $2 or even $0 (The New Zealand Ministry of Health 2013b) (The New Zealand Ministry of Health 2013c).

These safety nets do not protect against the cost of unfunded pharmaceuticals. However, agencies outside the public health system can in some cases assist with pharmaceutical costs. The Accident Compensation Corporation (a publically funded and administered insurance scheme) in some cases pays for pharmaceuticals to treat injuries resulting from accidents (New Zealand Accident Compensation Corporation (ACC) 2013). Publically funded disability payments are also available to assist with some medical costs (New Zealand Work and Income 2013). Perhaps as a result of these safety nets, fewer than 3% of New Zealanders face out-of-pocket pharmaceutical costs of US $1000 or more per year (The Commonwealth Fund 2013).

Ten percent of respondents had deferred collecting a prescription in the previous 12 months because of cost, a figure that is at the higher end of the range found in past studies (The Commonwealth Fund 2013; Jatrana and Crampton 2009). It is concerning this percentage hasn’t decreased despite Government efforts to improve access to primary healthcare. This group of respondents would have been more likely to collect their prescriptions if they had private insurance coverage for pharmaceuticals. However, past studies have shown that New Zealanders with lower incomes were more likely than to delay collecting their prescriptions (Jatrana and Crampton 2009). Our results show that this same group is less likely to have private insurance. Private insurance for pharmaceuticals is therefore unlikely to solve access problems in this group.

Another possible reason for consumers’ reluctance to pay for private coverage for pharmaceuticals is confidence the public health system will be able to provide the appropriate pharmaceuticals when needed. Overall, 67% of respondents were either confident or very confident in the public health system. Interestingly, respondents were more likely to have private insurance as their confidence in the public system decreased, but not more likely to be willing to pay for pharmaceutical cover through private insurance. This raises the intriguing possibility that despite the controversy PHARMAC generates, consumers may have come to trust the PHARMAC centred system can deliver what they need. Future work that explores this further may be beneficial.

To the best of our knowledge, this is the first study to examine New Zealand pharmacy consumers’ attitudes towards private insurance for pharmaceuticals. As such, it adds a further piece to the puzzle of how to provide New Zealanders with affordable access to new (and increasingly expensive medicines) while containing the total cost to the taxpayer.

## Conclusion

While the majority of pharmacy consumers believe having private insurance for pharmaceuticals would increase the availability and affordability of pharmaceuticals, willingness to pay was low. Slightly more than half were willing to pay extra for pharmaceutical coverage, of whom about two thirds were willing to pay $20 or less per year for such coverage. This may in part be due to the coverage provided by the public health system, in which again about two thirds of respondents were either confident or very confident.
